# Novel and efficient method for the reconstruction of adenoviruses through isothermal assembly and its potential applications

**DOI:** 10.3389/fmedt.2023.1095198

**Published:** 2023-01-26

**Authors:** Ke Wen, Matthew D. Resch, Ryan Mazboudi, Hannah Mulhall Maasz, Jose M. Galarza

**Affiliations:** TechnoVax, Inc., Elmsford, NY, United States

**Keywords:** adenoviral vectors, recombinant adenoviruses, rAdV14 and rAdV55, isothermal assembly, gene therapy, reporter adenoviruses

## Abstract

Adenovirus based vectors are useful tools for vaccine development, gene therapy, and oncolytic virotherapy. Here we describe a novel approach for the genetic engineering of any portion of the adenovirus genome and the reconstruction of either fully replication competent or defective virions. This innovative strategy is rapid, effective and suitable for the manipulation of the entire genome broadening the spectrum of potential applications for the adenovirus system. Our strategy involved insertion of restriction enzyme recognition sequences absent in the native virus into the termini of the adenovirus genome in order to facilitate recovery. These restriction enzyme sites, together with the two inverted terminal repeats and packaging sequences, were synthesized and then subcloned into the pBR322 vector. The remaining internal portion of the adenovirus genome was separated and amplified *via* PCR into six segments, of which groups of two were joined together by PCR and then subcloned into pBR322 plasmids. During the PCR reaction, an overlap of 30–40 bp was added to the termini of the adjacent fragments, allowing for the subsequent isothermal assembly and correct orientation of all fragments. This approach allows for the genetic modification of each genomic fragment according to the specific research goals, (e.g., deletion, substitution, addition, etc.) To recreate the entire viral genome, the four engineered fragments (each comprised of two adenovirus genomic sections) as well as the pBR322 backbone, were reassembled into a single construct utilizing an isothermal assembly reaction. Finally, the reassembled plasmid containing the entire genome was linearized and transfected into HEK293 cells to recover the complete reconstructed adenoviral vector. Using this approach, we have successfully generated two recombinant reporter adenoviruses, one of human adenovirus serotype 14 and another of serotype 55. The E3 region was replaced by the reporter genes (GFP and Luciferase) to visualize and track the recovery process. Subsequent infection of A549 cells with these reconstructed adenovirus vectors demonstrated that they were replication competent. This method shortens the viral reconstruction process because the one-step isothermal assembly requires less than 4 days, and recombinant adenovirus recovery occurs within 10 days. This new method allows for single or multiple genetic modifications within any portion of the viral genome and can be used to construct or manipulate any adenovirus whose complete genome sequence is known.

## Introduction

Inadequate delivery of genetic material is one of the main limiting factors for most *in vivo* gene and cancer therapy interventions. Viral vectors have occupied center stage in endeavors aimed at safely and effectively delivering prophylactic and therapeutic genes for the past two decades. Adenoviruses (AdVs) have been shown to be useful as vectors for gene transfer (1–3) due to their considerable advantages including: the ability to grow to high titers, the ability to infect both non-dividing and dividing cells, their diverse cell and tissue tropism, and their ability to be maintained as episomes in cells. Until recently, the predominant adenoviral vectors were derived from human adenovirus serotype 5 (HAdV5) (4). HAdV5, however, is found ubiquitously in the human population, infecting approximately 40%–60% of people and commonly leading to mild upper respiratory illnesses. This high prevalence has resulted in a significant level of HAdV5-specific neutralizing antibodies in the population (5), which decreases the therapeutic efficiency of AdV5-derived vectors while increasing their potential safety concerns and limiting clinical usefulness (6–8). Several approaches have been tried in an attempt to circumvent this limitation including the modification of the HAdV5 capsid genes (9–13) or the development of vectors derived from other adenovirus serotypes (4, 14–19). One important question that arises, however, is how to efficiently genetically manipulate the genomic sequences of the various adenovirus serotypes and then reconstruct new vectors with unique structural characteristics or novel genetic traits (e.g., fully replication competent, single cycle replication capable, defective, or genetically modified viruses).

Adenoviruses (AdVs) are non-enveloped, protein-based icosahedral capsid virions of approximately 70–90 nm in size that contain a non-segmented linear double stranded DNA genome ranging in length from −26 to −45 kb (20, 21). Suitable restriction enzyme sites are required to clone the entire adenovirus genome into a vector *via* sequential cloning of the resulting distinct genomic fragments. Due to the scarcity of suitable restriction enzyme sites in native adenovirus genomes, this process is limited to only a few strains. The most common method used to construct adenovirus vectors is based on a homologous recombination event between a shuttle vector and the adenovirus genome. This can take place in either mammalian cells (22) or in Escherichia coli (*via* the commercially available pAdEasy system) (23). However, both of these strategies need to be improved upon due to the low efficiency of homologous recombination exchange in mammalian cells, and significant time required to perform the complex processes involved in either procedure (22, 24–27). Although the efficiency of the homologous recombination process in E. coli is relatively high, the pAdEasy system currently available can only be used to construct AdV5-derived vectors. Therefore, to create diverse AdV vectors, a new and straightforward system must be advanced in order to genetically modify genes or sequences of any genome and then reconstitute distinctive shuttle vectors (28, 29).

Isothermal assembly is a seamless cloning method that allows for directional assembly of multiple DNA fragments in a single reaction without the use of restriction enzymes. This process was originally developed by Daniel G. Gibson and colleagues (30) and is utilized for constructing large-sized genomes. This method requires that the DNA fragments used contain unique overlapping sequences of approximately −40 bp between any two adjacent DNA segments, which then are accurately and efficiently glued together in a reaction containing an enzyme mix of a Taq DNA ligase, a mesophilic exonuclease, and a high-fidelity polymerase (31–35). One previously published study described a process in which seven amplified adjacent adenovirus fragments containing 15–20 bp overlapping segments were mixed together and underwent an isothermal assembly reaction with the resulting constructs then delivered to HEK293 packaging cells *via* electroporation in order to rescue the virus (36). We have attempted to replicate the procedure as outlined in the publication on several occasions but without success.

In this work we report a clear and novel strategy for the generation of adenoviral vectors *via* an isothermal assembly approach followed by virion recovery. Applying this method to adenoviral vector creation is a fast, flexible and efficient sequence-independent approach that not only expands the potential for reconstructing diverse viral genomes, but also reduces the time needed for their reconstruction. Furthermore, the method allows for the straightforward reworking of any adenovirus genes or sequences since the entire viral genome is partitioned into fragments that are then sub-cloned into several small plasmids where they can be engineered prior to reassembly. Finally, the vector is rescued utilizing a mammalian adenovirus packaging cell line [e.g., human embryonic kidney (HEK) 293 cells]. By implementing this strategy, we have developed several recombinant adenovirus serotypes, which are described here.

## Materials and methods

### Adenoviruses

Human adenovirus serotype 14 (HAdV14) (strain de Wit, ATCC, Cat#: VR-15, GenBank#: AY803294) was obtained from ATCC and the human adenovirus serotype 55 (HAdV55) (Strain AFMC 16-0011, GenBank#: KX494979) was kindly provided by Walter Reed Army Institute of Research (WRAIR), U.S. Department of Defense (DOD).

### Cell culture

HeLa cells (ATCC, Cat#: CCL-2) were maintained in Eagle's Minimum Essential Medium (EMEM) (ATCC, Cat#: 30-2003). AD293 cells (Agilent Technologies, Cat#: 240085) were maintained in Dulbecco's Modified Eagle Medium (DMEM) (Life Technologies, Cat#: 11995). A549 cells were maintained in ATCC-formulated F-12 K Medium (ATCC, Cat#: 30-2004). All culture media were supplemented with 10% FBS (Invitrogen, Cat#: 26140079), 1 unit of penicillin, and 1 μg/ml of streptomycin (Sigma, Cat#: P4333). All cells were cultured at 37 °C in 5% CO_2_.

### Purification of adenoviruses

HeLa Cells were seeded in a 175 cm^2^ flask and the following day, after reaching approximately −80% confluency, were infected with either HAdV14 or HAdV55. Culture media was changed every 3 days and at day 8 post infection, cells were washed twice with 25 ml of Tris-HCl (10 mM, pH 7.9), pelleted by centrifugation at 5,000 × g and then resuspended in 25 ml of Tris-HCl (10 mM, pH 7.9). Collected cells were then subjected to three freeze and thaw cycles (freeze at −80 °C and thaw at 37 °C in a water bath). Following the freeze thaw cycles the supernatant was collected by centrifugation at 5,000 × g for 10 min at room temperature (RT) and then loaded on top of a two-step CsCl density gradient, containing 5 ml of 1.25 g/cm^3^ CsCl in the upper layer and 2.5 ml of 1.4 g/cm^3^ CsCl in the lower layer prepared in a thin-walled 35-ml polypropylene centrifuge tube. The tubes were then spun at 27,000 rpm for 2 h at 10 °C in a Beckman SW28 rotor and the sharp, translucent band at the interface of the two CsCl layers was collected by piercing the side of tube with a needle and withdrawn *via* syringe. The density of the collected adenoviruses band was adjusted to 1.34 g/cm^3^ with a CsCl solution and then spun at 35,000 rpm for 16 h at 10 °C using a Beckman SW40TI rotor. Subsequently, the band located 1/3 of the way down from the top was collected, concentrated, and buffer exchanged with Tris-HCl (10 mM, pH 7.9) using a centrifugal filter (Millipore, Cat#: UFC910008). All CsCl solutions were prepared with 10 mM Tris-HCl at pH 7.9.

### Extraction of adenovirus genomes

A 400 μl volume of CsCl density gradient purified adenovirus was transferred into a 1.5-ml tube and treated for at least 3 h at 37 °C with 200 ug/ml of proteinase K (Invitrogen, Cat#: AM2546), 0.5% SDS and 500–1,000 units of RNase T1 (Roche, Cat# 109193). The mixture was then treated with a PCIA extraction solution (phenol: chloroform: isoamyl alcohol = 25: 24: 1) and the released adenovirus genome was subsequently precipitated with ethanol and then resuspended in DNAase-free UltraPure Distilled Water (Invitrogen, Cat#: 10977-015).

### Synthesis of part of the adenovirus genome and cloning into a pBR322 vector

**Figure 1:**
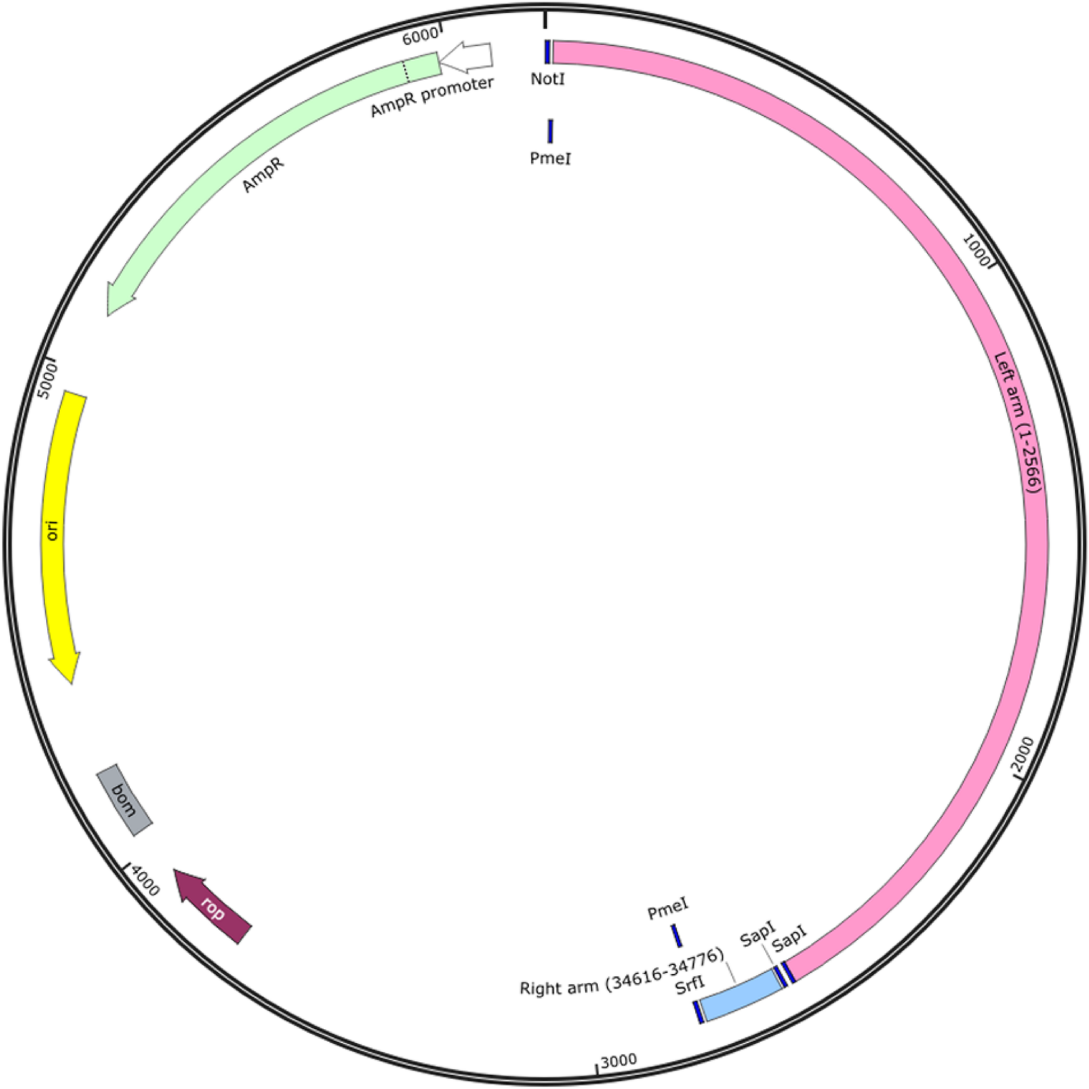
Strategy utilized for cloning the two adenovirus inverted terminal repeats (ITRs) and reassembling them with the remaining fragments of the adenovirus genome. The sequences of the left and right arms of the adenovirus inverted terminal repeats (ITRs), the packaging signal and specific restriction enzyme (RE) sites required for cloning or linearization were designed and chemically synthesized. The left arm contains the sequence from sites 1 to 2,566 and the right arm contains the sequence from sites 34,616 to 34,776 of the AdV55 genome, which are shared with the AdV14 genome. The NotI and SrfI RE sites were used for sub-cloning the synthesized sequences into the pBR322 vector. The PmeI sites were added to each end of the genome for linearization prior to rescue of the recombinant adenoviruses; the two SapI sites were added between the two ends of the ITR for the linearization of the left and right arms of the pBR322 vector prior to the assembly reaction with the other genome fragments. The resulting plasmid is identified as pBR322LR.

The low-copy number vector, pBR322 (New England Biolabs, NEB, Cat#: N3033S), was chosen for this study. A portion of this pBR322 vector, from sites 3 to 973, was subsequently replaced with a sequence containing multiple cloning sites (GCGGCCGCACTAGTGAATTCGGCCGGCCATTTAAATCGAATAGCCCGGGCATCGAACCTGCAGG) to allow for the sub-cloning of the adenovirus fragments. A sequence including the two-adenovirus inverted terminal repeats (ITRs) and the restriction enzymes sites needed for transfer, were chemically synthesized and positioned in a transport vector (Genscript, Piscataway, NJ). These fragments were then transferred into the pBR322 vector and the resulting construct was termed pBR322LR. Two additional SapI restriction enzyme sites were added between the two ITRs for the linearization of pBR322LR prior to the assembly reaction involving the other fragments of the adenovirus genome. A PmeI restriction enzyme site, absent in the genomes of both the AdV14 and AdV55 was added to each terminus for genome linearization prior to the recovery of the reassembled adenovirus virions. In order to transfer the previously synthesized left and right arm DNA sequences from the transport plasmid into the pBR322 vector, we utilized the NotI and SrfI sites added to each far end of these synthesized fragments. As illustrated in Figure 1, the configuration of the transport plasmid carrying the synthesized DNA contains the following features: the NotI site for sub-cloning into the pBR322 plasmid; the PmeI site for genome linearization prior to adenoviruses virion recovery; the left arm sequence; the SapI sites at each end of the left and right arms for plasmid linearization prior to the reassembly reaction with the remaining fragments; the right arm sequence; the SrfI site for sub-cloning into the pBR322 plasmid; and finally the PmeI sites needed for the linearization of the completely reassembled genome prior to adenovirus virion recovery. This chemically synthesized DNA can be utilized for work with either AdV14 and AdV55 due to their >99% sequence homology in this region. All restriction enzymes for these studies were purchased from New England Biolabs (NEB).

### Construction of three pBR322 vectors carrying distinct fragments of the adenovirus genome

**Figure 2:**
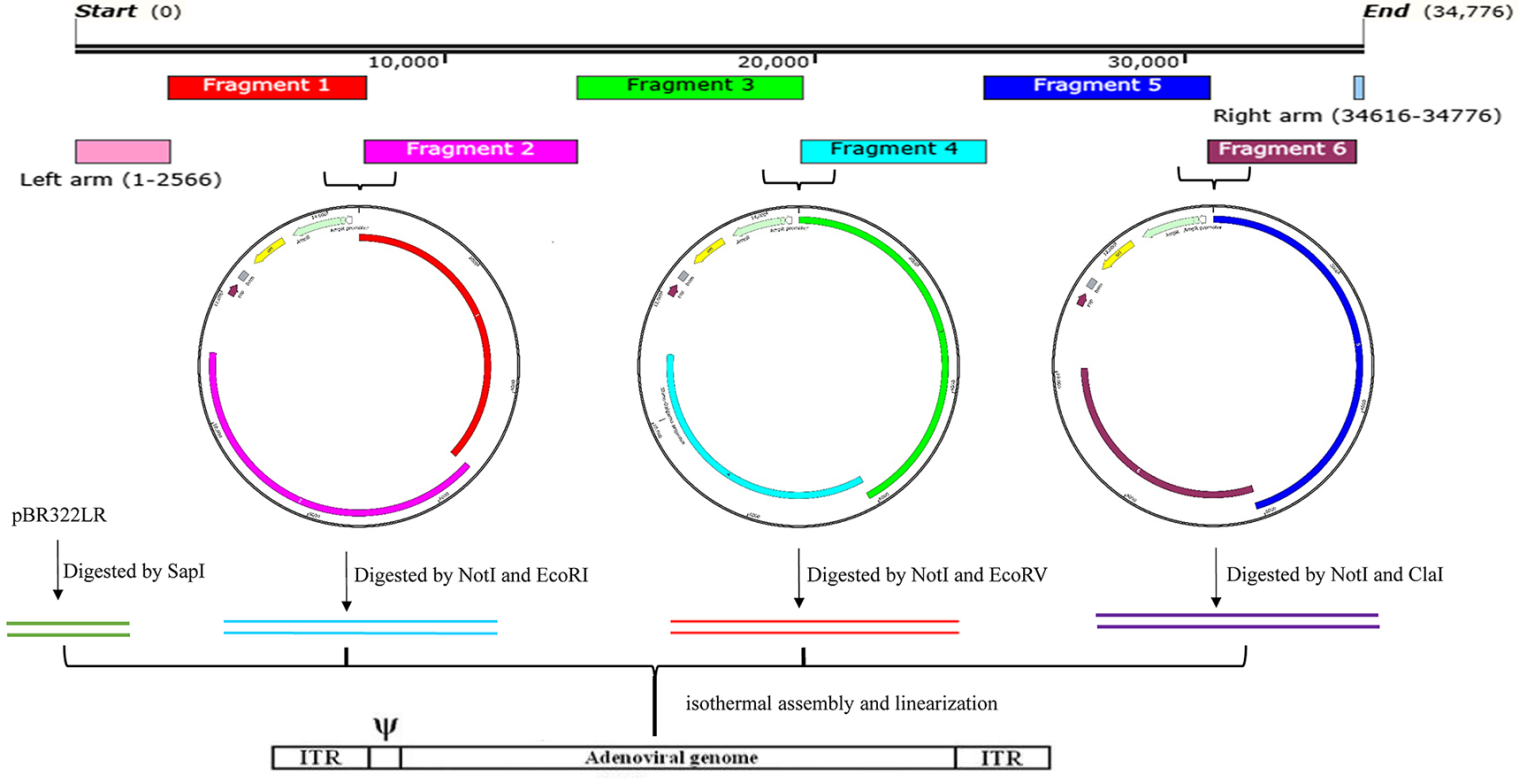
The overall strategy for the generation of recombinant adenoviruses. This includes, the layout of the AdV55 fragments, the cloning of two adjacent genomic DNA fragments into the pBR322 vector, the recovery of merged fragments by RE digestion, the isothermal reassembly of the whole genome and finally the linearization of the genome for the recovery of recombinant adenoviruses. The entire adenovirus genome, with the exception of the left and right arms contained in the pBR322LR, was divided and amplified into 6 fragments. Fragments 1 and 2, 3 and 4, or 5 and 6 were assembled as pairs by Gibson assembly with the Not/SbfI linearized pBR322 plasmid to generate three intermediate constructs (pBR322-1, 2; pBR322-3, 4; and pBR322-5, 6). These plasmids were then digested with two specific restriction enzymes (Not/EcoRI, NotI/EcoRV, and NotI/ClaI, respectively) to release the three adenovirus fragments which were subsequently used to reassemble, together with the SapI linearized pBR322LR, the complete adenovirus genome within pBR322. Since the E3 region was replaced by the luciferase and GFP expression cassette, the final plasmids containing the entire adenovirus genome were identified as: pBR322-recombinant AdV14 (pBR322-rAdV14) or pBR322-recombinant AdV55 (pBR322-rAdV55). Finally, these plasmids were linearized to rescue the recombinant adenoviruses that express the luciferase and GFP reporter genes.

The remaining adenovirus genome, with the exception of the two ITRs, was divided into 6 fragments (Fragment 1–6) and amplified by PCR using specific primers and a high-fidelity Q5 polymerase. The primer pairs specific for each fragment are listed in Table 1. The primers used to amplify the fragments adjacent to the pBR322 vector consisted of: 5′-25-bp pBR322 sequences, restriction enzyme site sequences, and 25-bp adenovirus sequences. The pBR322 sequences selected were: upstream- 5′AGGCCCTTTCGTCTTCAAGAATTGC3′- and downstream-5′ GGGCGACGCGAGGCTGGATGGCCTT-3′. The primers for any two adjacent adenovirus fragments were comprised of 25 bp of adenovirus sequences. The primer pair AdV-1-F and AdV-1-R were used for the amplification of Fragment 1, the AdV-2-F and AdV-2-R for Fragment 2, the AdV-3-F and AdV-3-R for Fragment 3, the AdV-4-F and AdV-4-R for Fragment 4, the AdV-5-F and AdV-5-R for Fragment 5, and the AdV-6-F and AdV-6-R for Fragment 6. Fragments pairs 1 and 2, 3 and 4, or 5 and 6 were assembled and cloned into a pBR322 vector previously linearized with restriction enzymes NotI and SbfI in order to construct three intermediate vectors (pBR322-1, 2; pBR322-3, 4; and pBR322-5, 6). The primer annealing sequences were carefully selected in order to allow for amplification of the genome fragments of either AdV14 or AdV55 due to the high degree of homology between these two viruses. The genome sequences for AdV14 and AdV55 are available on GenBank and are termed AY803294 and KX494979, respectively. The resulting three vectors were digested with the restriction enzymes, NotI/EcoRI, NotI/EcoRV, and NotI/ClaI, respectively (see Figure 2) to release the three fragments that were subsequently used, together with the SapI linearized pBR322LR vector, to reassemble the complete adenovirus genome now contained within the pBR322 vector.

**Table 1:** Specific primers for the amplification of the six adenovirus fragments.

ADV-1-F	AGGCCCTTTCGTCTTCAAGAATTGCGGCCGCTGAGGTGGTAATAGATACTCCAGACAAGACA
ADV-1-R	ATCGGCTCTCATCCTCGCACAGAAAGAC
ADV-2-F	AGGTCAGGAAGAGTCTTTCTGTGCGAGGATG
ADV-2-R	AAGGCCATCCAGCCTCGCGTCGCCCGAATTCGAGGGCGCAAATGAGCAAACGG
ADV-3-F	AGGCCCTTTCGTCTTCAAGAATTGCGGCCGCAAGGGGCAACCCGTTTGCTCATTT
ADV-3-R	CATTGGAATAAAGGAAACTTCGCCATAGATTGG
ADV-4-F	CTTCAAGCCAATCTATGGCGAAGTTTCC
ADV-4-R	AAGGCCATCCAGCCTCGCGTCGCCCGATATCATATCTTGCACGCCTGCCGCACAAAG
ADV-5-F	AGGCCCTTTCGTCTTCAAGAATTGCGGCCGCACGGCTTTGTGCGGCAGGC
ADV-5-R	GTTGGGAAGAGGGAAGTGAGGTGCTG
ADV-6-F	TTCTCCCAGCAGCACCTCACTTCCC
ADV-6-R	AAGGCCATCCAGCCTCGCGTCGCCCATCGATTGCAAGTTAAGCGGATGTGACGTCCCG

### Construction of a recombinant adenovirus whose E3 region is replaced with a luciferase-GFP expression cassette

The primer pair (AdV-5-F' and AdV-5-R') (Table 2) was used to amplify Fragment 5 from AdV14 or AdV55, which was subsequently digested with NotI and SrfI and ligated into the pBR322 vector cut with same restriction enzymes. The resulting vector, identified as pBR322-5, was then amplified using the primers (AdV14forLG-F and AdV14forLG-R for AdV14, and AdV55forLG-F and AdV55forLG-R for AdV55) (Table 2) which resulted in the deletion of the E3 region, that comprised the sequence from 27,333 to 30,601 and from 27,832 to 30,593 of the AdV14 and AdV55, respectively. This deleted portion of the viral genome was subsequently replaced by a luciferase and GFP expression cassette. The backbone of a pMCS-luciferase vector (Thermo Scientific, Cat#: 16146) was utilized to construct the vector, pCMV-luciferse-SV40-GFP, which is able to express both the luciferase and GFP reporter genes. This plasmid was subsequently digested with EcoRV (three sites in the vector) to release the luciferase and GFP expression cassette that was then reassembled with a previously linearized pBR322-5 vector carrying the adenovirus Fragment 5. The resulting vector, identified as pBR322-5LG, was tested *via* transfection to verify expression of the reporter genes, and then amplified with primers (AdV-5-F and AdV-5-R) in order to reassemble it together with Fragment 6 and the pBR322 vector. Since the E3 region was replaced with the luciferase and GFP expression cassette, the resulting vectors were identified as pBR322-recombinant AdV14 (pBR322-rAdV14) or pBR322-recombinant AdV55 (pBR322-rAdV55).

**Table 2:** Primers for cloning fragment 5 into the pBR322 vector and replacement of the E3 region with the luciferase and GFP expressing cassette.

ADV-5-F’	CGATAAGCGGCCGCACGGCTTTGTGCGGCAGGC
ADV-5-R’	AATAGCGCCCGGGCGTTGGGAAGAGGGAAGTGAGGTGCTG
ADV14forLG-F	ATCGCTCTTCGTAAAACCTCTACAAATGTGGTAGATAAATCACTTACTTAAAATCAGC
ADV14forLG-R	CGCGTGCTCTTCGTATATCTGGCCCGTACATCGGATTTACCTTCGATAGTAATCC
ADV55forLG-F	ATCGCTCTTCGTAAAACCTCTACAAATGTGGTAGATAAAAATGATTAATAAAAAATCACTTAC
ADV55forLG-R	CGCGTGCTCTTCGTATATCTGGCCCGTACATCGGATTTTTTAAACCAATACCACAAC

### Ligation, isothermal assembly and transformation

Ligation reactions were carried out with T4 DNA Ligase (NEB, Cat#: M0202), using a 1:3 molar ratio of vector to insert, and incubated at 16 °C overnight. Prior to transformation the ligation mixture was heat-inactivated at 65 °C for 10 min. Isothermal assembly was performed using the NEBuilder HiFi DNA Assembly Master Mix (NEB, Cat#: E2621) with an amended protocol. Briefly, a molar ratio of 1: 2–3 of vector to fragment was used to enhance assembly efficiency. The assembly mixture was incubated in a thermal cycler for 30 min (2 fragments) or 90 min (≥3 fragments) at 50 °C with 55 °C for the lid temperature. A 5 μl of ligation or assembly mixture was subsequently used to transform 100 μl of either E. coli strain SURE 2 Supercompetent Cells (Agilent Technologies, Cat#: 200152) or MAX Efficiency Stbl2 Competent Cells (Life Technologies, Cat#: 10268019). Subsequently, 900 μl of SOC medium was added to an E. coli transformation mix and further cultured for 90 min at 33 °C and shaken at 200 rpm. The transformation mixture was then spread onto ampicillin-containing LB plates and incubated for 16 h at 33 °C. Multiple colonies were selected and individually culture by incubating for 20 h at 33 °C and shaking at 200 rpm. The transport vector from Genscript (Piscataway, NJ) and pBR322LR were both transformed into SURE 2 Supercompetent Cells and several bacterial colonies were selected for analysis to verify the integrity of the plasmids through subsequent restriction enzyme digestion and sequencing. The other vectors were transformed into MAX Efficiency Stbl2 Competent Cells and several bacterial colonies selected for plasmid testing to verify their accuracy and integrity *via* restriction enzyme digestion.

### Transfection of AD-293 cells with linearized pBR322-rAdV14 or pBR322-rAdV55 vectors to rescue the adenovirus recombinants

The pBR322-rAdV14 and pBR322-rAdV55 vectors were linearized with PmeI, purified *via* a PCIA procedure, ethanol precipitated, and then resuspended in DNAase-free UltraPure distilled water. AD293 cells were seeded onto a single 6-well plate one day prior to transfection with the linearized pBR322-rAdV14 or pBR322-rAdV55 vectors. Transfections were performed following the procedure outlined in the Agilent ViraPack Transfection Kit (Agilent Technologies, Cat#: 200488), Briefly, AD293 cells with a confluency of −70% were washed twice with PBS and then incubated for 30 min. in culture media containing MBS and 25 µM of chloroquine. During this time, the linearized vectors were mixed with solution I and solution II and incubated for 15 min at RT, and the mixture was then added to cells and followed by a 3 h incubation at 37 °C. The culture medium was then replaced with a fresh preparation containing 25 µM chloroquine and incubated for an additional 7 h. Finally, the culture medium was changed to complete culture medium and cells incubated for 10 days with fresh culture medium replenishment every 2–3 days. The progress of the transfection procedure and virus recovery was monitored daily by fluorescent microscopy examination. Images of cells expressing GFP were taken at days 7 and 10 post-transfection. Forty microliters of culture supernatant were collected at days 0, 1, 3, 5, 7 and 10 post-transfection for the assessment of luciferase activity which was performed in duplicated assays for each sample (20 µl reaction). Cells were collected at day 10 post-transfection and resuspended into 2 ml Tris-HCl (10 mM, pH 7.9) and the recombinant adenoviruses were extracted following three freeze/thaw cycles and a clarifying step *via* centrifugation as described in the Section ‘Purification of adenoviruses’.

### Measurement of luciferase activity

The luciferase activity in a 20 µl culture supernatant sample was measured using the BioLux Gaussia Luciferase Assay Kit as follows: (1) the GLuc assay solution was prepared by adding 50 µl of BioLux GLuc Substrate and 800 µl of BioLux GLuc Stabilizer to 5 ml of BioLux GLuc Assay Buffer; (2) the solution was mixed thoroughly and incubated at RT for 25 min; (3) the luminometer was then set to allow for 2–10 s of integration; (4) samples were pipetted (20 µl per well) into a 96-well plate; (5) the assay solution was added to all wells (50 µl per well) and subsequently samples were incubated at room temperature for 35–40 s and then measured in a luminometer.

### Examination of infectious activity of recombinant AdV14 (rAdV14) and rAdV55

A549 cells were seeded in one 6-well plate and upon reaching −80% confluency were infected with 100 µl of recombinant AdV14 (rAdV14) or recombinant AdV55 (rAdV55) purified as outlined in the above section. The progression of infection was monitored daily by fluorescent microscopy and images collected at days 1–2 post-infection.

## Results and discussion

In this study, we describe a novel procedure for the generation of recombinant adenoviruses displaying unique genetic properties. We have constructed two recombinant adenoviruses (rAdV14 and rAdV55) that are able to express the luciferase and green fluorescent protein (GFP) reporter genes which are positioned within the deleted E3 region of the adenovirus genome. To generate these recombinant viruses and demonstrate the versatility of this approach, we have carried out the following steps: the two-adenovirus inverted terminal repeats (ITRs) and important restriction enzyme sequences were synthesized in order to facilitate the recovery of these reengineered adenoviruses. In order to maintain the sequence integrity of the ITRs, both the transport vector and pBR322LR (Figure 1) were transformed into SURE 2 Supercompetent cells, which lack pathways that catalyze the rearrangement and deletion of nonstandard secondary and tertiary structures, allowing for the cloning of sensitive DNA sequences. The two PmeI sites were added to each end of the AdV14 and AdV55 genomes, which do not contain this restriction enzyme site naturally, in order to enable their linearization and subsequent recovery of the virions. Other unique restriction enzyme sites could also be selected for this purpose provided that they are absent in the genome. PCR primers were designed to incorporate at each end of two adjacent fragments an overlap of 30–40 bp to allow for the correct assembly and orientation of all segments.

**Figure 3:**
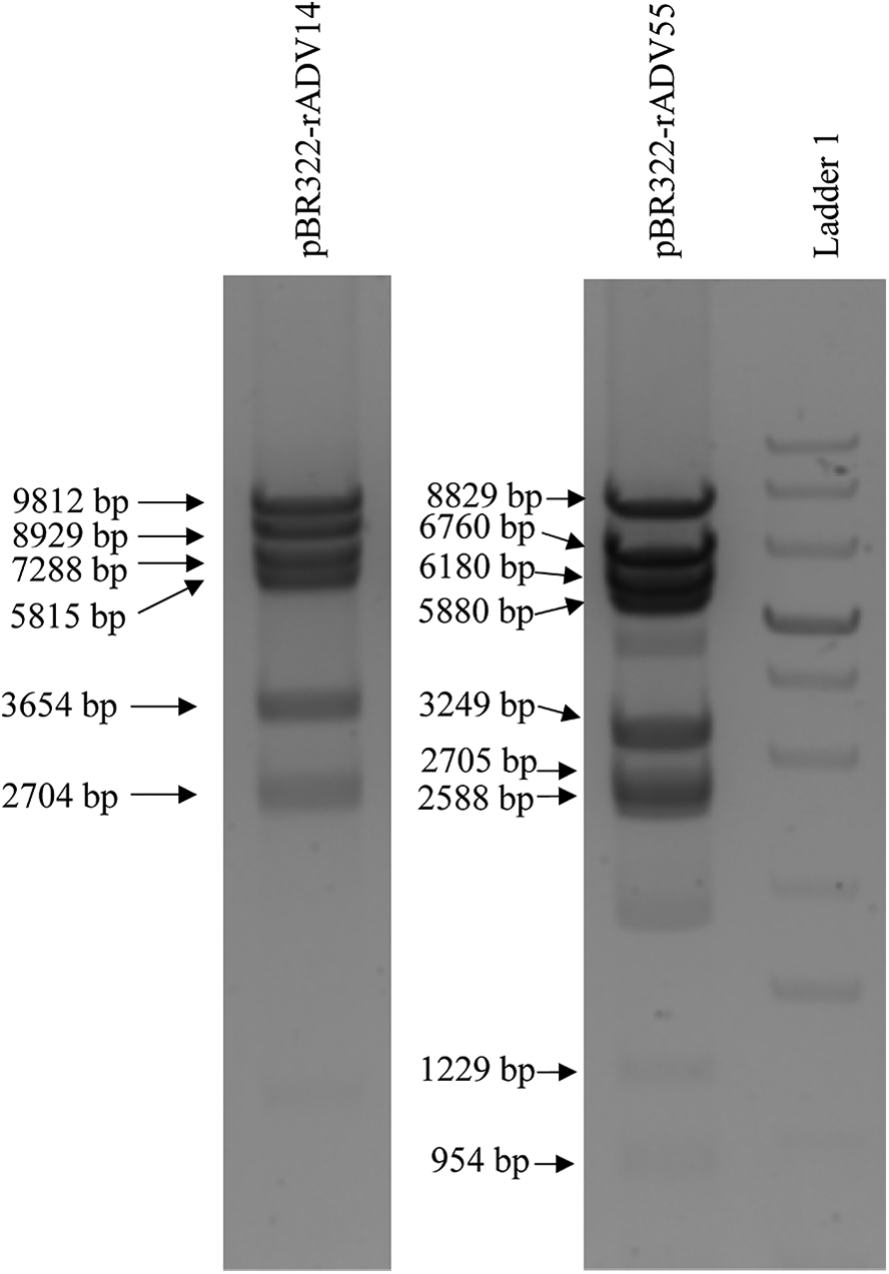
Restriction enzyme analysis of the pBR322-rAdV14 and pBR322-rAdV55 constructs. Analysis of the vector was performed by digesting 2.5 ug of the pBR322-rAdV14 DNA with the restriction enzymes EcoRI, NheI and ClaI. This digestion analysis resulted in 6 segments with sizes of 9,812, 8,929, 7,288, 5,815, 3,654 and 2,704 bp. The restriction pattern of the pBR322-rAdV55 when digested with NheI, PmeI, XbaI and ClaI resulted in shows 9 segments with sizes of 8,829, 7,660, 6,180, 5,880, 3,249, 2,705, 2,588, 1,229 and 954 bp. These patterns correspond to those expected for correct constructs. Ladder 1 is a GeneRuler 1 kb Plus DNA Ladder (Thermo Scientific, Cat#: SM1331).

In order to avoid the introduction of random mutations during the PCR reactions, the high-fidelity Q5 polymerase (which has >280 times higher fidelity than Taq polymerase) was utilized for all of the amplification procedures, which results in ultra-low error rates. In addition, the size of each the adenovirus fragment is approximately 6 kb (Figure 2), which allows for the Q5 polymerase to amplify these fragments with optimal performance. The four relatively large genomic fragments were released from the vectors *via* digestion with specific restriction enzymes, which circumvents the need to amplify fragments with large sizes (Figure 2). All released fragments were subsequently reassembled into the vectors, pBR322-rAdV14 and pBR322-rAdV55, *via* an isothermal assembly reaction and the accuracy of the resulting constructs was verified by restriction enzyme digestions as shown in Figure 3.

Since any adenovirus genome can be divided into several fragments, this approach is suitable for manipulation of any region of various adenovirus genomes.

**Figure 4:**
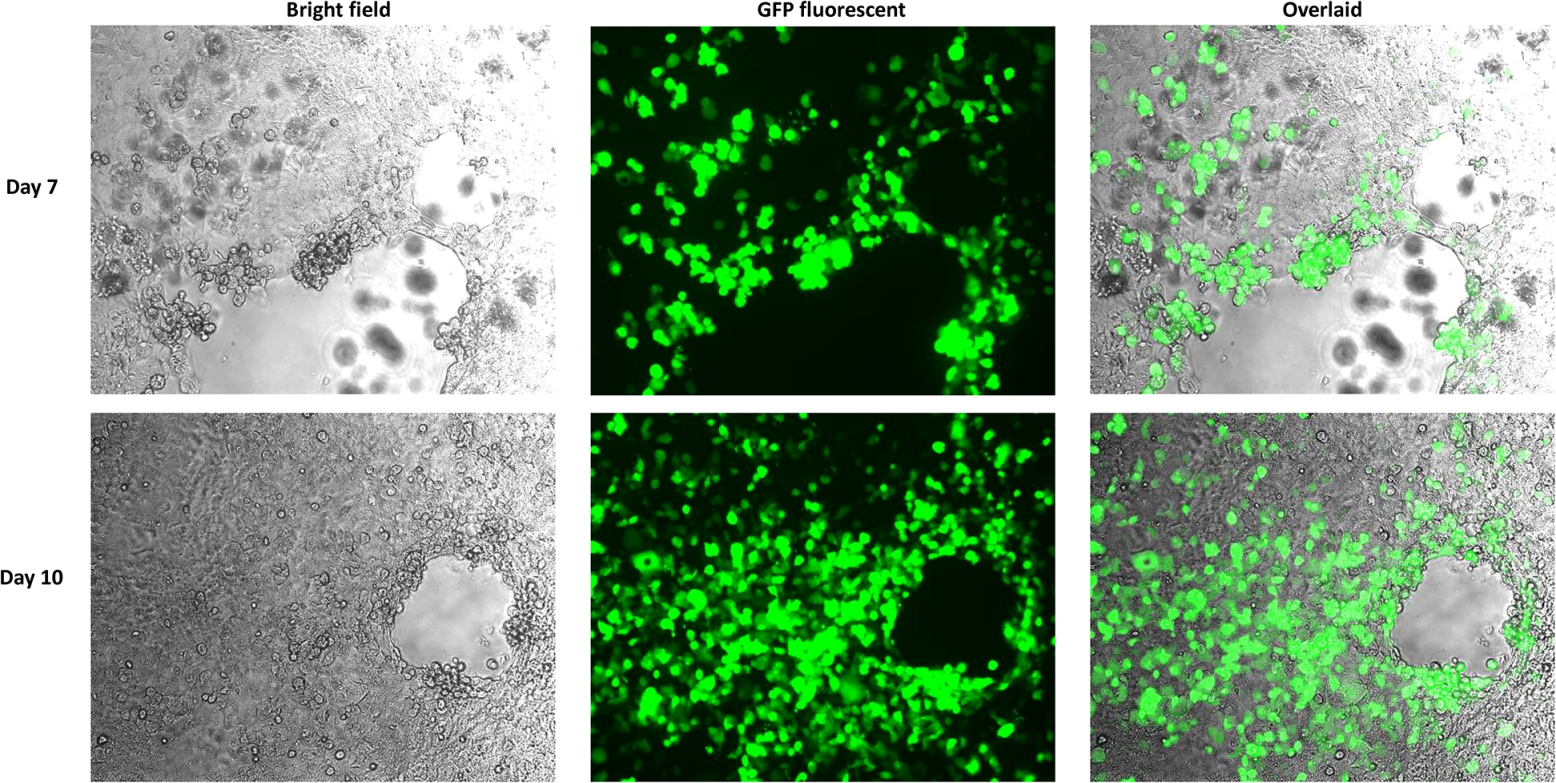
GFP fluorescent imaging of AD293 cells on days 7 and 10 post transfection with the linearized rAdV14 genome. AD293 cells were seeded on a 6-well plate one day prior to transfection with the linearized rAdV14. The culture medium was replaced with a fresh preparation on days 3, 5, and 7, and GFP fluorescent images were taken 7 and 10 days post-transfection. The cytopathic effect caused by the adenovirus replication (bright field) and the expression of the GPF (fluorescent) are colocalized as can be seen in this overlaid image.

**Figure 5:**
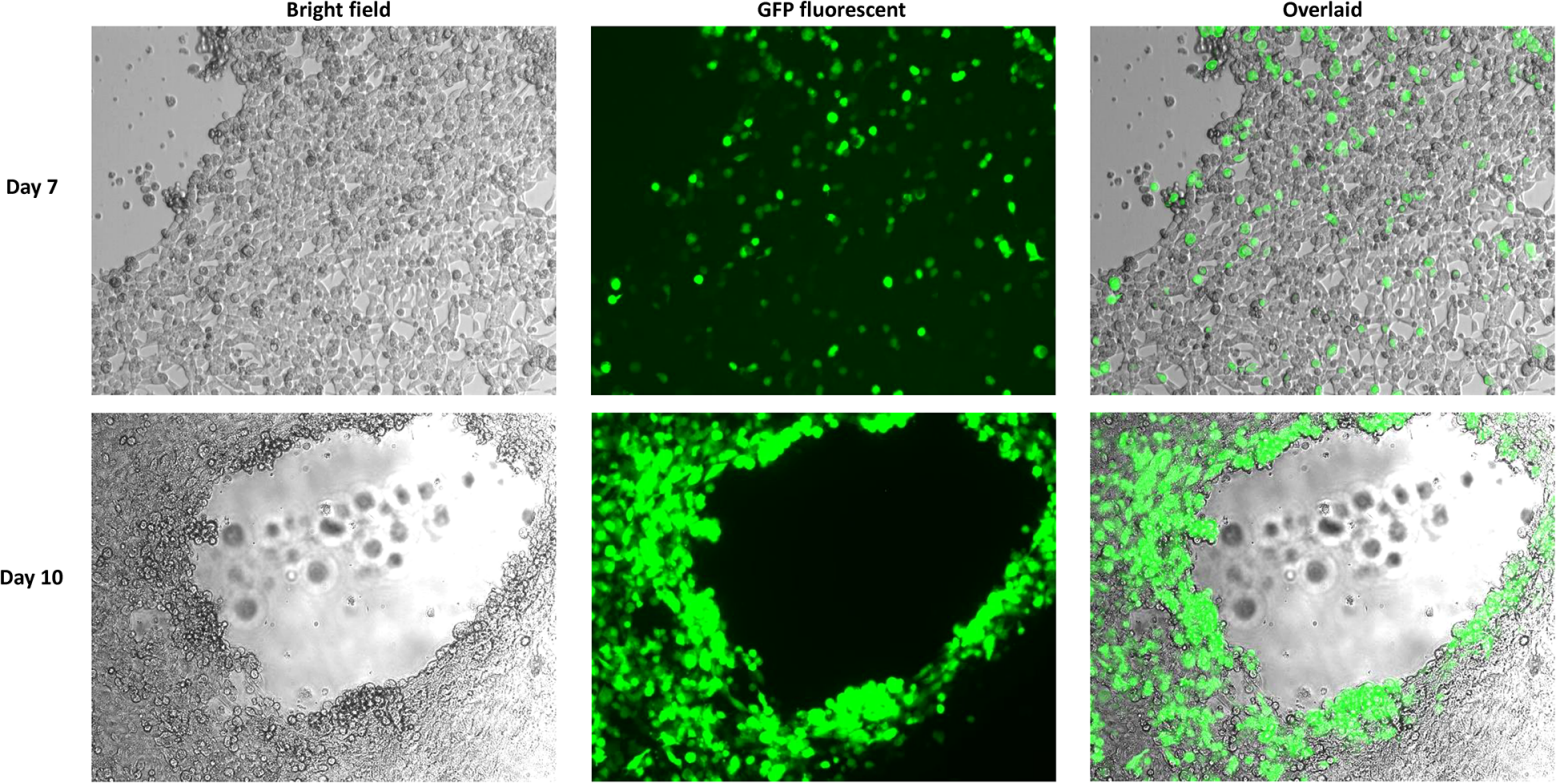
GFP fluorescent imaging of AD293 cells 7 days and 10 days post transfection with the linearized rAdV55 genome. AD293 cells were seeded on a 6-well plate one day prior to transfection with linearized rAdV55. See the legend for Figure 4 for a description of the treatment and imaging of cells.

**Figure 6:**
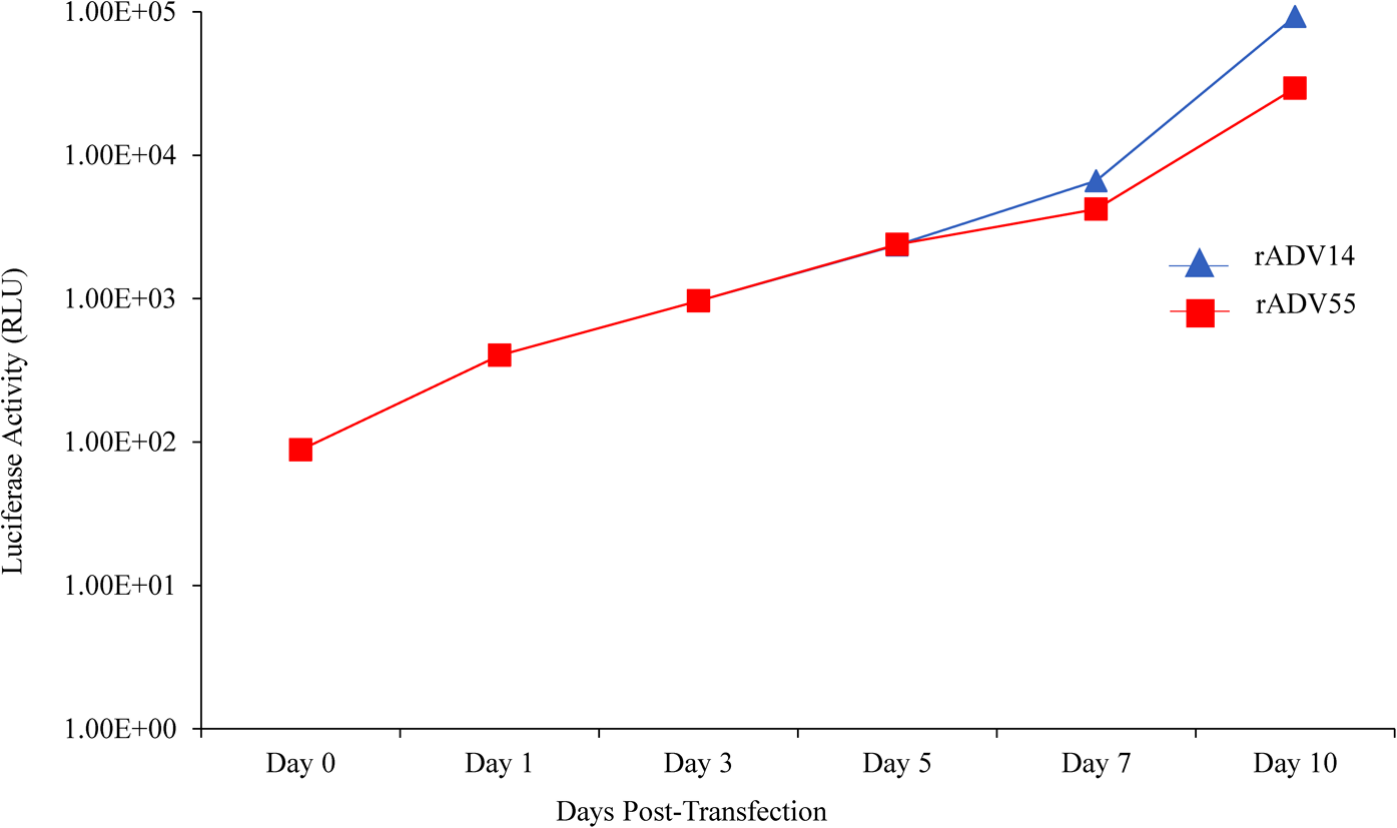
Detection of luciferase activity in the supernatant of AD293 cells following transfection with the linearized rAdV14 or rAdV55 genomes. Forty microliters of cell culture supernatant were collected at days 0, 1, 3, 5, 7 and 10 post transfection to measure the luciferase activity. See the legends for Figures 4, 5 for a description of the treatment of the cells.

Reassembled vectors pBR322-rAdV14 and pBR322-rAdV55 were cut to release the genome and used to transfect AD293 cells for virus recovery. The detection of high levels of green fluorescent protein expression at both days 7 and 10 post transfection, indicated that the recovery of the rAdV14 or rAdV55 was very efficient (Figures 4, 5). In addition to the detection of GFP, the luciferase activity induced by the rAdVs in cell culture supernatants was demonstrated post transfection over time (Figure 6). A linear increase of luciferase activity occurred on days 1–7 post transfection, suggesting an incremental replication of rAdV14 and rAdV55 during this period, however this activity accelerated on days 7–10 post transfection, suggesting that at least 7 days are required for the adenovirus recovery procedure. If adenovirus rescue does not occur or if recovered virus is intended for pharmaceutical application, all amplified fragments can be sequenced to ensure that no unexpected mutations have been introduced.

**Figure 7:**
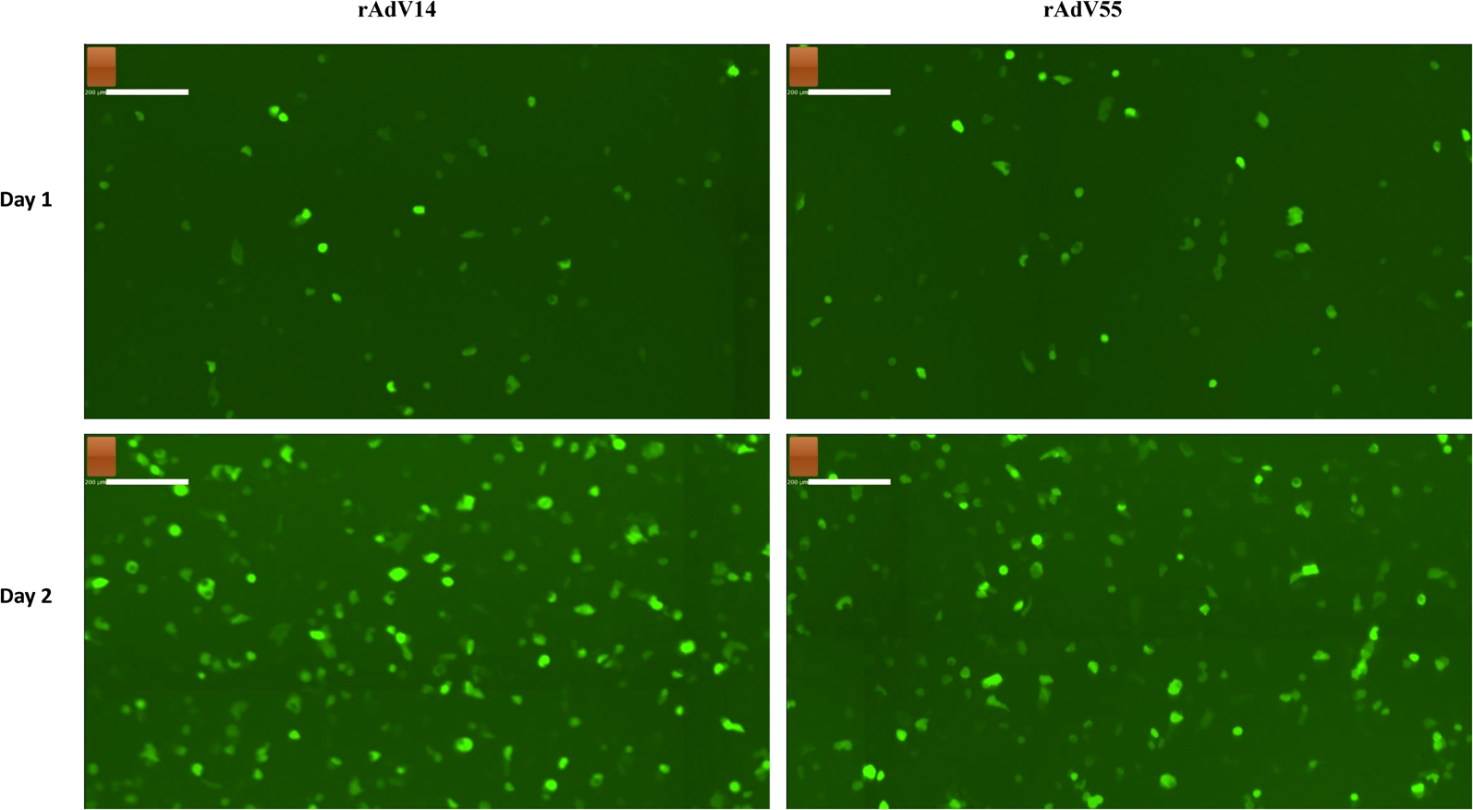
GFP fluorescent imaging of infected A549 cells by rAdV14 and rAdV55. A549 cells were seeded in one 6-well plate and after reaching −80% confluence the following day were infected with recombinant AdV14 (rAdV14) or recombinant AdV55 (rAdV55). The progress of the infection was monitored daily by fluorescent microscopy and images were taken at days 1–2 post infection.

Since A549 cells do not express the adenovirus E1 gene products, they are only able to support the growth of replication-competent (or wildtype) adenoviruses. Therefore, we examined the infectivity of the rAdV14 and rAdV55 in this cell line. As shown in Figure 7, there was a significant level of GFP expression in the A549 cells on days 1 and 2 post infection with both recovered adenoviruses, suggesting that they are fully replication-competent and infectious in a non-complementary cell line.

In this paper, we describe a new method for the construction of recombinant adenoviral vectors based on an isothermal assembly approach, independent of the process that requires the creation of a shuttle vector and subsequent sub-cloning of genes into this vector, thus avoiding the complex procedures of homologous recombination and selection. This novel procedure is rapid and efficient, providing flexibility to not only genetically engineer any region of the viral genome, but also to allow for work with distinct adenovirus serotypes. Since the adenovirus genome can be divided randomly into multiple fragments, it is possible to introduce minor or major genetic changes within specific genes or regulatory sequences.

This procedure requires less than two weeks to isolate adenovirus genomes, generate adenoviral molecular clones, and construct recombinant adenoviral vectors. Additionally, this method can be used to construct any adenovirus serotype with a known sequence. The isothermal assembly and generation of recombinant adenoviral vectors described in this work offers a new strategy for creating novel adenoviral vectors that can be used as carriers for vaccines, therapeutic genes, or oncolytic drugs, among other potential uses, expanding the utility of adenoviruses as biological tools.

## Data Availability

The datasets presented in this study can be found in online repositories. The names of the repository/repositories and accession number(s) can be found in the article/Supplementary Material.
